# Adenocarcinoma of the Lung With Metastasis to the Small Intestine: Case Report

**DOI:** 10.1002/rcr2.70511

**Published:** 2026-02-17

**Authors:** Moeko Ota, Naofumi Hara, Ryo Kato, Kiriko Onishi, Yusuke Fujioka, Fumie Onishi, Hisaaki Tanaka, Nobukazu Fujimoto

**Affiliations:** ^1^ Department of Respiratory Medicine Okayama Rosai Hospital Okayama Japan; ^2^ Department of Gastroenterology Okayama Rosai Hospital Okayama Japan; ^3^ Department of Medical Oncology Okayama Rosai Hospital Okayama Japan

**Keywords:** anaemia, capsule endoscopy, ipilimumab, nivolumab, small intestine metastasis

## Abstract

A 40‐year‐old man presented with dyspnea and dizziness. He was found to have severe anaemia and computed tomography revealed a mass in the right upper lobe of the lung. Despite a positive test for faecal occult blood, the source of bleeding could not be identified. The patient was diagnosed with adenocarcinoma of the lung, cT3N2M0 stage IIIB. Initially, he received chemotherapy consisting of cisplatin and docetaxel and thoracic radiotherapy. However, a few days after the initiation of chemoradiotherapy, his anaemia worsened. Capsule endoscopy revealed multiple ulcers and erosions in the intestinum jejunum. A biopsy using double‐balloon endoscopy revealed poorly differentiated tumour cells with similar characteristics to those in the lung. The clinical stage was modified to T3N2M1c stage IVB. The patient was treated with chemo‐immunotherapy consisting of carboplatin, paclitaxel, ipilimumab, and nivolumab. He is currently doing well after completing 2 years of treatment.

## Graphical abstract

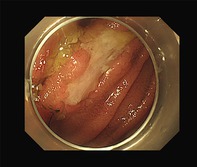

## 1 Introduction

Gastrointestinal metastasis of lung cancer is rare and difficult to detect [1]. Such metastases are often identified only after causing severe complications, such as massive bleeding, bowel obstruction or perforation. We report a case of non‐small cell lung cancer with severe anaemia due to metastasis to the small intestine detected by capsule endoscopy. The examination aided in clinical staging and the selection of appropriate treatment.

## 2 Case Report

**FIGURE 1:**
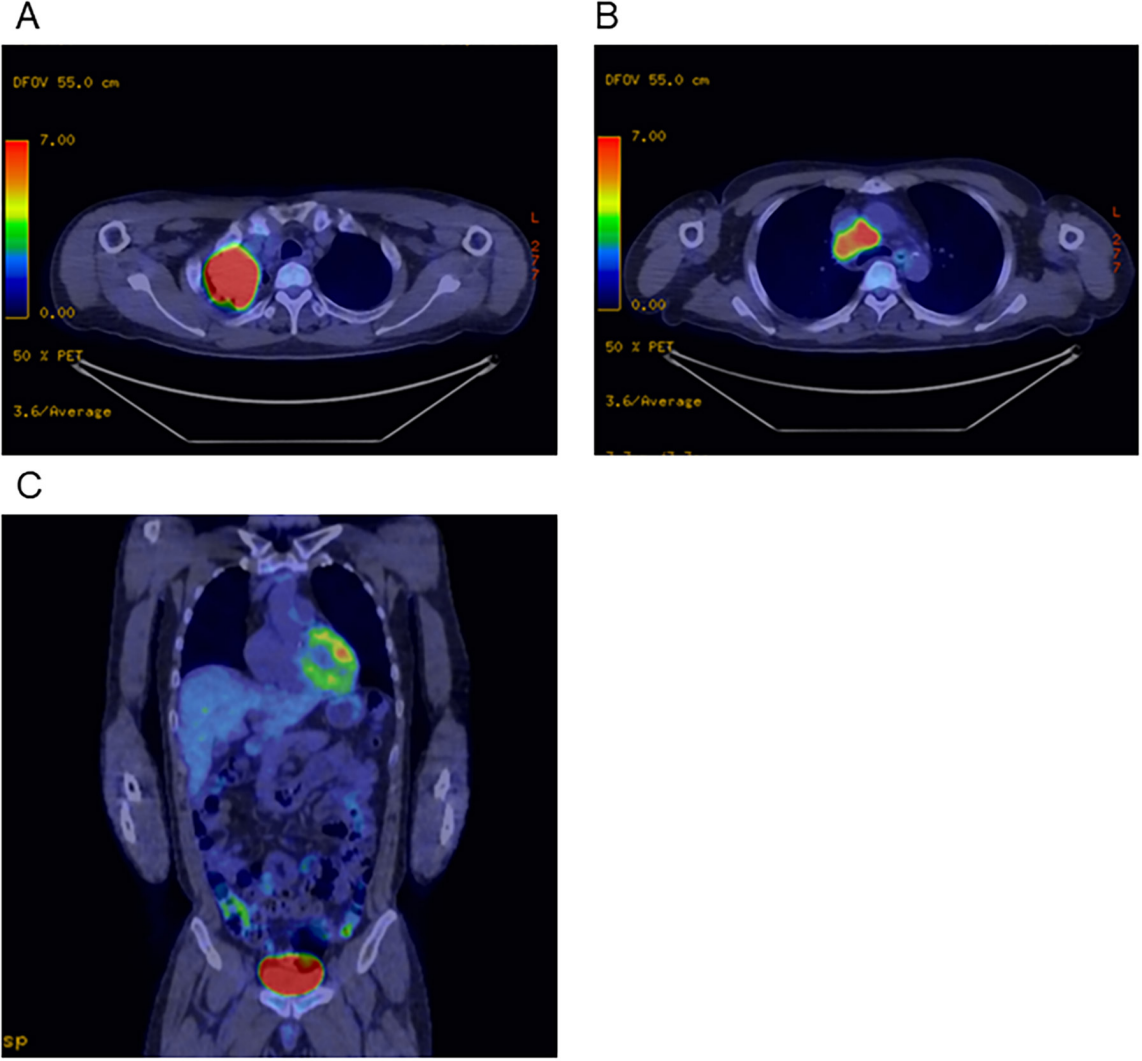
(A, B) Chest PET‐CT images revealing increased FDG uptake in the mass in the right upper lobe and the mediastinal lymph nodes. (C) PET‐CT did not reveal any FDG uptake in the abdomen. FDG, fluorodeoxyglucose; PET‐CT, positron emission tomography/computed tomography.

A 40‐year‐old man was referred to our hospital with dyspnea and dizziness persisting for 2 weeks. He had a 28 pack‐year smoking history but no other medical history. Laboratory tests showed severe anaemia with a haemoglobin level of 4.6 mg/dL and haematocrit decrease of 16.5%. The mean corpuscular volume was 75.3 fL, mean corpuscular haemoglobin 21.0 pg, ferrum 22 μg/dL and ferritin 16.54 ng/mL. White blood cell and platelet counts were within normal ranges. The faecal occult blood test was positive. Computed tomography (CT) revealed a mass 63 mm in diameter in the right upper lobe of the lung. Positron‐emission tomography (PET) showed fluorodeoxyglucose (FDG) uptake in the pulmonary mass and mediastinal lymph nodes. No abnormal FDG uptake was observed in abdominal organs (Figure 1). Bronchoscopic biopsy resulted in a diagnosis of adenocarcinoma, and the patient was negative for driver mutations. Neither upper esophagogastroduodenoscopy nor large bowel endoscopy detected any source of bleeding or neoplastic lesions. Based on these staging work‐ups, the patient was diagnosed with adenocarcinoma of the lung, cT3N2M0 stage IIIB. The anaemia was attributed to temporal small intestinal bleeding and subsequent iron deficiency anaemia.

**FIGURE 2:**
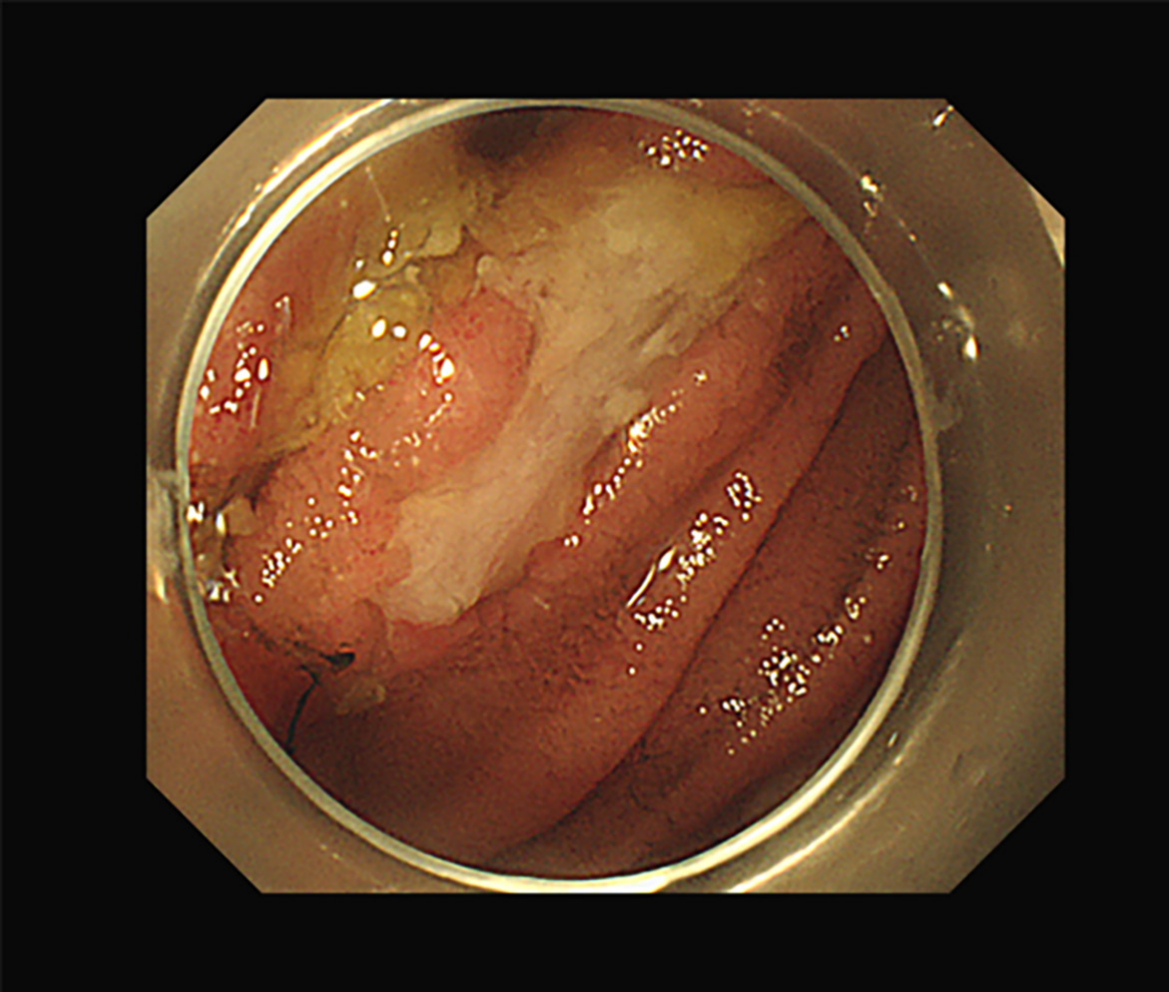
Double‐balloon endoscopy revealed multiple ulcers and erosions in the intestinum jejunum.

Initially, the patient received chemotherapy consisting of cisplatin and docetaxel and thoracic radiotherapy. His anaemia was treated with a red blood cell transfusion and iron supplementation. However, a few days after the initiation of chemoradiotherapy, the anaemia worsened. The retest for faecal occult blood was positive. Capsule endoscopy was performed and revealed multiple ulcers and erosions in the intestinum jejunum (Figure 2). A biopsy was subsequently obtained using double‐balloon endoscopy and revealed poorly differentiated tumour cells. The immunohistochemical characteristics were as follows: The primary tumour of the lung was all negative for TTF‐1, Napsin A, p40, CDX2 and SATB‐2, and the tumour of the intestinum jejunum was positive for CK7, weak positive for CDX2, few positive for p40, and negative for TTF‐1, CK20 and SATB‐2. Based on these results, we diagnosed the tumour in the intestinum jejunum as metastasis of lung cancer. At this point, the patient was diagnosed with adenocarcinoma of the lung with metastasis to the small intestine. The clinical stage was modified to T3N2M1c stage IVB. The treatment approach was switched to systemic chemotherapy consisting of carboplatin, paclitaxel, ipilimumab and nivolumab after 1 month of the initial treatment.

The patient responded well to the chemo‐immunotherapy; both the tumour in the right lung and the mediastinal lymph nodes significantly decreased in size, and his anaemia improved. He is currently doing well after completing 2 years of treatment.

## 3 Discussion

Gastrointestinal metastasis from primary lung cancer is uncommon and difficult to detect in a clinical setting because of a lack of sensitivity of imaging modalities, such as CT and PET‐CT [2]. In the current case, the patient had severe anaemia at his initial presentation. Gastrointestinal bleeding was strongly suspected, but upper esophagogastroduodenoscopy and large bowel endoscopy did not detect any source of bleeding or neoplastic lesions. Furthermore, neither contrast‐enhanced CT nor PET‐CT detected any distant metastasis of the lung cancer. Small bowel metastasis was not suspected initially because patients with small bowel metastases often have metastases at other sites [3]. As a result, the patient was misdiagnosed with stage III disease and treated with curative chemoradiotherapy. Prolonged anaemia motivated us to further examine the small intestine, and capsule endoscopy and double‐balloon endoscopy finally detected metastasis to the small intestine that caused bleeding anaemia Capsule endoscopy has a higher diagnostic rate for small intestinal tumours than contrast‐enhanced CT [4] and should be considered in cases with unexplained anaemia or in which metastasis to the small intestine is suspected.

Gastrointestinal metastasis often leads to life‐threatening complications, such as bleeding, intestinal obstruction, and perforation. The prognosis is poor, as half of those diagnosed with gastrointestinal metastasis have been reported to have died within 3 months [1]. However, immunotherapy successfully controlled intestinal bleeding from metastasis in a recent report [5]. In the current case, a combination of chemotherapy and immune checkpoint inhibitors resulted in a favourable and prolonged response. Thorough identification of the cause of anaemia led to the selection of appropriate treatment.

In conclusion, we reported a case of adenocarcinoma of the lung with severe anaemia due to metastasis to the small intestine. Gastrointestinal metastasis should be considered when unexplained anaemia is present in patients with lung cancer. A thorough examination should be conducted to detect the anaemia.

## Author Contributions

Moeko Ota, Naofumi Hara, Ryo Kato, Kiriko Onishi, Yusuke Fujioka, Fumie Onishi, Hisaaki Tanaka contributed to Conceptualisation, Investigation, Writing – Original draft preparation. Nobukazu Fujimoto contributed to Investigation and Supervision.

## Ethics Statement

Submission was approved by the ethics committee of Okayama Rosai Hospital.

## Consent

The authors declare that written informed consent was obtained for the publication of this manuscript and accompanying images and attest that the form used to obtain consent from the patient complies with the Journal requirements as outlined in the author guidelines.

## Conflicts of Interest

N.F. received honoraria from Ono Pharmaceutical. Other authors declare no conflicts of interest.

## Data Availability

The data that support the findings of this study are available on request from the corresponding author. The data are not publicly available due to privacy or ethical restrictions.
